# Tolerance Induction Strategies in Vascularized Composite Allotransplantation: Mixed Chimerism and Novel Developments

**DOI:** 10.1155/2012/863264

**Published:** 2012-12-24

**Authors:** David A. Leonard, Duncan A. McGrouther, Josef M. Kurtz, Curtis L. Cetrulo

**Affiliations:** ^1^Transplantation Biology Research Center, Massachusetts General Hospital, Boston, MA 02129, USA; ^2^Department of Plastic and Reconstructive Surgery Research, University of Manchester, Manchester, UK; ^3^Biology Department, Emmanuel College, Boston, MA, USA

## Abstract

Since the start of the clinical vascularized composite allotransplantation (VCA) era over a decade ago this field has witnessed significant developments in both basic and translational research. Transplant tolerance, defined as rejection-free acceptance of transplanted organs or tissues without long-term immunosuppression, holds the potential to revolutionize the field of VCA by removing the need for life-long immunosuppression. While tolerance of organ and vascularized composite transplants may be induced in small animal models by a variety of protocols, only mixed-chimerism-based protocols have successfully bridged the gap to preclinical study and to clinical trial in solid organ transplantation to date. In this paper we review the mixed-chimerism approach to tolerance induction, with specific reference to the field of VCA transplantation, and provide an overview of some novel cellular therapies as potential adjuvants to mixed chimerism in the development of tolerance induction protocols for clinical vascularized composite allotransplantation.

## 1. Introduction

The challenges of transplantation have engaged plastic and reconstructive surgeons since the early years of the specialty [1]. During the past decade, since the start of the clinical vascularized composite allotransplantation era, progress in this field has accelerated, with significant developments in both basic and translational research [2, 3]. 

Currently, vascularized composite allotransplantation remains dependent on long-term immunosuppression in order to prevent graft rejection. While modern immunosuppressive medications are effective in controlling acute rejection, they have little impact on chronic rejection; an incompletely understood phenomenon, observed in all branches of solid organ transplantation, which leads to a progressive decline in transplant function [4]. The requirement for life-long immunosuppression, and the attendant risks and side effects of current regimens which include metabolic disorders, renal impairment, infectious complications and an increased risk of tumor development, present a major cause for concern in the treatment of conditions which are not, in contrast to solid organ transplantation, immediately life threatening. 

Induction of donor specific transplant tolerance, defined as the specific absence of a destructive immune response to a transplanted tissue in the absence of immunosuppression, is a primary goal of transplantation research, and holds the potential to avoid the risks posed by long-term immunosuppressive regimens. Tolerance would also overcome chronic rejection, the impact of which on VCA has not yet become clear, but which should be considered a real possibility as follow up continues into the long term [5, 6]. Unfortunately, while tolerance of a variety of organ and composite tissue transplants may be reliably induced in small animal models, translating these findings to large animal preclinical models and clinical trials has proved challenging, and to date only protocols utilizing lymphohematopoietic mixed chimerism have proved successful at inducing tolerance across genetic disparities at these levels [7]. The term mixed chimerism has been subjected to a widely encompassing definition, ranging from donor hematopoietic stem cell engraftment with stable long-term multilineage contribution, to regimens using donor bone marrow infusion to achieve transient mixed chimerism that may or may not be followed by a state of microchimerism. These various states of chimerism and the relative role they play in the induction and maintenance of tolerance of transplanted tissues have been demonstrated to vary across models and target organs [8].

These results provide proof of concept for clinical transplantation tolerance, and it can be hoped that further development of tolerance protocols will overcome the stringent challenge posed by composite transplants including skin, and lead to development of clinically applicable protocols for VCA tolerance. In this paper we will review progress in the development of mixed-chimerism-based tolerance protocols, and outline encouraging areas of research with potential for development of novel alternatives to current immunosuppressive regimens in vascularized composite allotransplantation.

## 2. Mixed Chimerism and Transplant Tolerance

In immunological terms, a chimera is an individual in whom a proportion of the hematopoietic cell population can be identified as originating from another individual. This may occur naturally, as in the case of Owen's freemartin cattle [9], but is most usually the result of hematopoietic cell transplantation [10]. In the context of tolerance induction, it is also important to differentiate between full and mixed chimerism. Full chimerism describes the complete replacement of an individual's hematopoietic system with donor cells. This is commonly seen following treatment for hematological malignancy, but is associated with reduced immunocompetence and a significant risk of graft versus host disease (GvHD) [11]. Mixed chimeras, as the name suggests, possess a mixture of recipient and donor hematopoietic cells. Mixed chimerism requires less stringent conditioning of the recipient, maintains immunocompetence and has a lower risk of graft versus host disease, and as such is preferable as a potential tolerance induction strategy [12].

The use of hematopoietic cell transfer in production of chimeras and hence induction of donor specific tolerance has been known for many years, and this approach remains at the forefront of attempts to develop clinically applicable tolerance induction strategies for all forms of surgical transplantation. Interest in this approach has been maintained by reports of patients accepting organ transplants without chronic immunosuppression, having previously received bone marrow transplants from the same donors [13]. Early experimental protocols in mice relied on myeloablative conditioning and complete reconstitution with a donor hematopoietic stem cell graft in order to achieve engraftment and chimerism [14]; however such protocols carry significant morbidity and a risk of impaired immunocompetence which would not be considered acceptable outside the field of hematological malignancy. The development of non-myeloablative conditioning regimens achieving engraftment and mixed chimerism with significantly reduced morbidity represents a key step in the search for clinically relevant tolerance induction regimens and has been demonstrated in small animals [15], large animals [16], and in clinical protocols [17]. 

**Figure 1:**
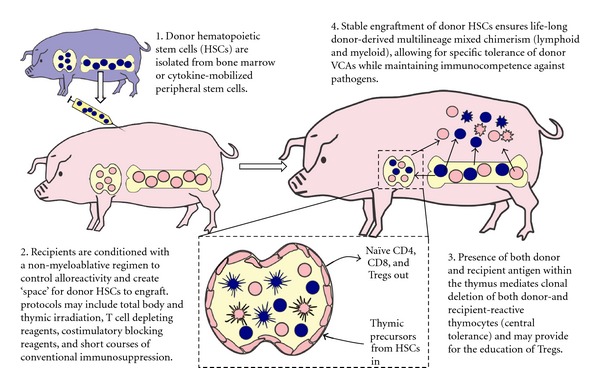
Mechanisms of tolerance in mixed chimerism.

It has been proposed that donor stem cell engraftment, resulting in maintenance of donor cell lineages within the recipient, provides continued presentation of donor antigen, facilitating donor-specific tolerance through the same central and peripheral mechanisms responsible for self-tolerance (Figure 1) [13]. The presence of antigen presenting cells of both donor and recipient origin within the thymus will facilitate clonal deletion of both donor and recipient-reactive thymocytes, as reviewed by Sykes [12]. It is presumed that alloreactive thymocytes escaping deletion will be controlled by T regulatory cells, which may be educated either in the thymus or in the periphery within transplanted tissues. 

Progressive clonal deletion of peripheral T cells has been identified as an important mechanism [18]. However, non-deletional mechanisms have also been shown to play a role, as donor specific tolerance can be demonstrated before clonal deletion is complete [19–21]. The balance of regulatory and deletional mechanisms in tolerance is complex, but there is evidence in mild regimes, achieving low level chimerism, that regulatory mechanisms predominate [13, 22]. Regulatory mechanisms appear to retain an important role during the induction of tolerance by more stringent regimes, but this gradually declines with time, as progressive deletion of donor-reactive T cells continues [21, 23].

Interestingly, the requirement for the establishment of stable mixed chimerism may not be applicable for the long-term rejection free survival of various organs and tissues. In both preclinical studies in nonhuman primates, and the MGH clinical trial, transient mixed chimerism was found to be sufficient for induction of renal allograft tolerance [24, 25]. It is hypothesized that T regulatory cells, educated during the period of chimerism, are responsible for maintenance of tolerance in this scenario. Pronounced FoxP3^+^ T cell infiltration in the absence of signs of inflammation or tissue damage was observed within the kidneys tolerated by recipients in the nonhuman primate studies, suggesting local regulation of the immune response within the donor organ [24]. In contrast to these findings of organ tolerance despite loss of detectible chimerism, Leventhal and colleagues have recently reported establishment of durable chimerism and kidney transplant tolerance, without graft versus host disease. Non-myeloablative conditioning and transplantation of an enriched hematopoietic stem cell graft in combination with an infusion of graft-facilitating cells, composed primarily of plasmacytoid precursor dendritic cells, achieved chimerism and permitted weaning of immunosuppression in five of eight patients [26].

In contrast to kidneys, studies in large animal recipients of bone marrow and vascularized composite allografts suggest that these transplants may have more stringent requirements. A series of studies in MGH Miniature Swine indicate that while tolerance of the musculoskeletal components of vascularized composite allografts may be induced by protocols achieving transient chimerism, tolerance of skin requires engraftment of donor hematopoietic stem cells and persisting mixed chimerism [27–29]. Skin has long been regarded as the most robust test of transplant tolerance [30]. Initial studies utilized a heterotopic hind limb model, with or without skin paddle, transplanted between MHC-matched, minor antigen mismatched animals, treated with a 12 day course of Cyclosporine A. All animals in both groups accepted the musculoskeletal components of the transplanted limb long-term. Those in the skin-free transplant group subsequently received frozen donor split thickness skin grafts, which rejected [31]. Recipients of skin-bearing transplants demonstrated prolonged skin survival, in one case to 180 days, but in all cases epidermal rejection eventually occurred [27]. This state of tolerance to one organ or tissue, while simultaneously rejecting another, has a long historical record, as it was first described by Billingham and Silvers and termed “split tolerance” [32]. These findings are consistent with previous studies demonstrating that skin is consistently more prone to rejection than other tissues, but that primarily vascularized skin appears to enjoy a survival advantage over conventional skin grafts. While tissue specific antigens are often offered as a potential explanation for the difficulty in achieving skin acceptance, a definitive skin specific antigen is yet to be identified, and other factors including graft size, the skin immune system and the inflammatory milieu resulting from a period of relative ischemia in the absence of primary vascularization have all been implicated [33–35].

Subsequent studies addressed the important step of transplantation across major histocompatibility barriers, once again utilizing the skin-bearing heterotopic limb model. In this series, the musculoskeletal components of the limb were once again uniformly tolerated, across both single haplotype and full class I and class II MHC barriers in recipients of hematopoietic stem cell transplantation using either cytokine-mobilized peripheral blood mononuclear cells or bone marrow cells. Prolonged skin survival to between 35 and 50 days was observed but tolerance of skin was not demonstrated [28]. Animals receiving cytokine mobilized cells received a significantly higher dose of hematopoietic cells than those receiving bone marrow, and demonstrated detectable albeit progressively declining peripheral blood mixed chimerism, while those receiving bone marrow did not. Regardless, these studies further illustrate that while kidney tolerance may be achieved by similar protocols in the context of both transient and long-term chimerism, induction of tolerance of skin components of VCAs will require more robust induction mechanisms. The relationship between chimerism and tolerance has often been controversial [36, 37], and in this model it appears that stable chimerism is not necessary for tolerance of musculoskeletal components of the allografts. 

In 2009 Horner et al. published a preliminary report of the successful induction of tolerance to skin across a major histocompatibility barrier in the MGH miniature swine model in which stable mixed chimerism was established using a non-myeloablative conditioning regimen and cytokine mobilized hematopoietic stem cells. Following confirmation of donor stem cell engraftment, primarily vascularized skin flaps and conventional skin grafts were transplanted, and in this experiment one animal demonstrated tolerance of its flap for over 300 days of follow up. This tolerance was robust, as demonstrated by the acceptance of a subsequent donor split thickness skin graft placed 124 days following the original skin flap [29]. Recently, similar results in a canine model of vascularized composite allotransplantation have been reported by Mathes et al., with long-term graft survival and stable mixed chimerism in a MHC-matched, minor antigen mismatched model [38]. These studies support the hypothesis that engraftment of donor hematopoietic stem cells, and persisting mixed chimerism are required for tolerance of skin in these large animal models.

## 3. Cellular Therapies in Mixed Chimerism and VCA Tolerance

Considerable reductions in the toxicity and morbidity of conditioning regimens have been achieved since the initial studies utilizing myeloablative protocols, although the majority of these have been described in small animal models. Thus, achieving mixed chimerism while minimizing the adverse effects of conditioning remains a challenging balance and a variety of novel strategies have been investigated as potential adjuncts in an effort to enhance engraftment and mitigate complications such as GvHD.

### 3.1. T Regulatory Cells

Regulatory cells have been extensively studied in the context of transplantation tolerance. The existence of a population of lymphocytes capable of suppressing immune responses was first described 40 years ago, and shortly thereafter these cells were demonstrated to facilitate tolerance of non-self antigens in a murine skin transplant model, demonstrating the potential importance of these cells in achieving tolerance of the skin component of VCA [39, 40]. The characterization and diverse functions of the canonical CD4^+^CD25^+^FoxP3^+^ Treg cell population has been extensively reviewed [41]. The ability of these cells to enhance engraftment following allogeneic bone marrow transplantation was described by Joffre et al. who subsequently demonstrated that CD4^+^CD25^+^FoxP3^+^ T regulatory cells could prevent both acute and chronic rejection of skin and cardiac allografts [42, 43]. 

Direct evidence for cellular regulation by CD4^+^CD25^+^ T cells has been demonstrated in some murine models of bone marrow transplantation. Bigenzahn and colleagues found that depletion of CD25^+^ cells at the time of bone marrow transplantation and costimulatory blockade (anti-CD154 and CTLA4Ig) blocked development of tolerance, but that late depletion of CD25^+^ cells failed to abrogate tolerance, demonstrating that, in this model, the role of Tregs was most prominent during induction rather than maintenance phases [44]. Pilat and colleagues recently demonstrated that recipient T regulatory cells, administered in conjunction with anti-CD40L mAb and CTLA4Ig costimulatory blockade and a short course of Rapamycin, could achieve engraftment and stable multilineage chimerism, and subsequent skin tolerance, following radiation-free conditioning and conventional dose bone marrow transplantation in a fully mismatched murine model [45]. Interestingly, Rapamycin was found to be an essential component of this protocol, which is consistent with other studies finding that Rapamycin facilitates selective expansion of T regulatory cells while inhibiting clonal proliferation of effector cells [46].

Issa and colleagues in Oxford have reported the development of a humanized mouse model, in which they have extensively investigated the potential of human Tregs as a tolerance induction strategy for transplantation. They recently demonstrated the ability of these cells to prevent transplant arteriosclerosis (a hallmark of chronic graft rejection), and uniquely, to induce tolerance to human skin allografts [47]. There is also evidence that, in the context of bone marrow transplantation, donor CD4^+^CD25^+^ cells may protect against acute graft versus host disease (GvHD) [48, 49]. Taken together and in combination with the work done by many other groups, these experiments are certainly encouraging, but further work is required to refine the specificity of Treg markers, and to provide evidence of efficacy in large animal models prior to considering the therapeutic application of T regulatory cells in clinical composite tissue allotransplantation. 

### 3.2. Dendritic Cells

The traditional view of dendritic cells (DCs) as potent inducers of immune reactivity has been augmented in recent years by the recognition that, as specialized antigen presenting cells, they have the ability to facilitate immunologic tolerance [50, 51]. It is logical that dendritic cells exert their tolerogenic effects through interaction with T cells, and indeed, they have been shown to suppress CD4^+^ and CD8^+^ T cell proliferation [52], and to control activation and function of CD4^+^CD25^+^Foxp3^+^ Tregs [53].

The accepted paradigm states that immature dendritic cells (characterized by low expression of cell surface MHC II and costimulatory molecules) induce tolerance upon interaction with T cells, while mature dendritic cells (high expressers of MHC II and costimulatory molecules) induce an effector response from T cells [54]. However, it has been demonstrated that dendritic cells of both immunogenic and tolerogenic phenotypes may be directed toward a change of phenotype by ligation of specific cell surface receptors [55]. Therefore, while the existence of functionally distinct subsets of dendritic cells is being continuously defined and expanded (reviewed in [56]), it seems likely that the distinction between tolerogenic and immunogenic roles does not lie along simple divisions between subsets, and that dendritic cells demonstrate functional plasticity, although the discriminating factor in this plasticity remains controversial. 

Attempts have been made to exploit the tolerogenic potential of dendritic cells in animal models of transplantation with some success. Tolerance of cardiac transplants has been achieved in rodent models following administration of donor allopeptide-pulsed DCs in combination with a short course of antilymphocyte serum [57]. Long term survival of skin allografts and hind-limb composite tissue allotransplants, has also been demonstrated, in some cases with demonstrable expansion of Tregs [58–62]. In the context of mixed chimerism protocols, it has been demonstrated in a murine model that cotransplantation of bone marrow with immature dendritic cells lead to engraftment and stable multilineage chimerism without cytoreductive conditioning and with no evidence of graft versus host disease. These chimeras accepted cardiac allografts long-term, and while skin tolerance was not achieved, challenge skin grafts did enjoy significantly prolonged survival [63]. 

 There have been some early studies in nonhuman primates which have demonstrated the presence of tolerogenic dendritic cells with the ability to modulate the T cell response to alloantigens [64], and, it has been demonstrated that dendritic cells are able to induce tolerance of model antigens and facilitate expansion of T regulatory cells in human volunteers [65, 66]. These findings point to the possibility of “negative cellular vaccines” as a potential route to tolerance in composite tissue allotransplantation, but further preclinical work is required [67]. 

### 3.3. Mesenchymal Stem Cells

Mesenchymal stem cells (MSCs) are a component of the bone marrow stroma, and play homeostatic roles important for hematopoiesis through synthesis of numerous cytokines and growth factors including granulocyte colony stimulating factor (G-CSF), stem cell factor (SCF), Flt-3 ligand and members of the interleukin family [68, 69]. MSCs have been shown to lack expression of costimulatory molecules and consequently to have limited capability for stimulating alloreactive T cells [70–72]. Furthermore, MSCs have been demonstrated to possess immunological inhibitory potential, suppressing proliferation in mixed lymphocyte cultures and prolonging skin graft survival in a rodent allotransplant model [73, 74].

The natural physiologic role of MSCs within bone marrow stroma has been exploited in the successful treatment of GvHD in patients following bone marrow transplantation [75], and recently to facilitate engraftment and induction of tolerance to limb composite tissue allografts in animal models. Using a rat model, Pan and colleagues performed hind-limb allotransplantation after having induced chimerism 30 days previously with a regime of antilymphocyte serum, rapamycin, 3 Gy total body irradiation, bone marrow cells and ex vivo expanded MSCs. Rapamycin was continued for 100 days, and following cessation of treatment, animals exhibited stable chimerism, tolerated their transplanted limb for greater than 100 days without any exogenous immunosuppression, and showed no evidence of GvHD [76]. 

MSCs have also been reported to be a useful adjunct to bone marrow transplantation for tolerance induction in a large animal model. In an outbred miniature swine model, Kuo and colleagues demonstrated survival of heterotopic hind-limb allotransplants for greater than 200 days in animals treated with irradiation, bone marrow transplantation, 28 days of cyclosporine, and three doses of donor MSCs (each of 1 × 10^7^ cells, on days 7, 14, and 21 post limb transplant). Interestingly, while no signs of GvHD were observed, the same regime in the absence of MSCs resulted in a maximal allograft survival of 57 days and symptoms of severe GvHD ultimately resulting in death [77]. This study provides proof of principle that MSCs may be an effective adjunct to bone marrow transplantation in tolerance induction, and taken together with previously reported clinical use of MSCs for treatment of GvHD, indicates that this may be a useful and interesting avenue for further research in composite tissue allotransplantation. 

## 4. Conclusions and Future Directions

The shared history of reconstructive and transplant surgery have, over the past 15 years, witnessed a new chapter with the emergence of vascularized composite allotransplantation as a viable option for the treatment of patients with severe, complex extremity and craniofacial defects for which the outcomes of conventional reconstructive techniques remain suboptimal. While clinical data demonstrate the short to medium term efficacy of these procedures, the decision to prescribe life-long immunosuppression in the treatment of non-life-threatening condition remains an ethical dilemma. 

The induction of donor specific tolerance holds the potential to avoid both the risks of life-long immunosuppression and to prevent chronic rejection. A clinically applicable tolerance strategy for vascularized composite allotransplantation would fundamentally alter the risk-benefit analysis for potential recipients and could expand availability of these procedures to patients currently considered high risk or unsuitable candidates, for example those requiring restoration of congenital anomalies or following oncological resection. Skin remains a particularly stringent test of any transplant tolerance protocol, and while tolerance of skin has been reported in preclinical studies further work is required to demonstrate that this can be reliably achieved by a clinically applicable protocol.

While there is encouraging evidence from small animal models for a wide variety of tolerance strategies, mixed chimerism is the only approach to prove successful in large animal studies, or to reach clinical trials in organ transplantation, and is the established frontrunner in preclinical studies of composite tissue tolerance. While it has been shown clinically that transient mixed chimerism and other immunomodulatory approaches are sufficient for induction of tolerance of other organs, and can play an important role in minimization of immunosuppression for VCA [25, 78], to move toward the goal of true tolerance and immunosuppression free VCA acceptance, stable mixed chimerism appears to have the most promise at this time. Despite a steady reduction in the toxicity of experimental regimens, the morbidity associated with the conditioning regimes required to permit engraftment of hematopoietic stem cells remains a concern. Despite success in small and now large animal models translation to clinical application remains challenging. In addition to the combined kidney and bone marrow transplantation studies reported by several centers [25, 26], recent work by Bolaños-Meade et al. [79] in the field of HLA mismatched bone marrow transplantation demonstrates progress toward establishment of chimerism across MHC barriers with a low incidence of GvHD suggesting that the goal of inducing VCA tolerance with mixed chimerism remains highly possible. In this paper we have reviewed a number of adjuvants to the mixed chimerism approach, which appear to have the potential to enhance engraftment and to mitigate complications such as GvHD. Some of these strategies have already been tested in preclinical models with encouraging results, and it can be hoped that further translational studies will result in development of safe, effective tolerance induction protocols for clinical trial in vascularized composite allotransplantation.
